# Supplementary material for: Data-In-situ Computing with One-Pixel-Multiple-Memristor Architecture for Neuromorphic Sequential Vision
Source: Nat Commun. 2026 Mar 19;17:4244. doi: 10.1038/s41467-026-70860-y (PMC13168392; doi:10.1038/s41467-026-70860-y)
Supplement: Supplementary file 1 — Supplementary Information [file 41467_2026_70860_MOESM1_ESM.pdf]

Supplementary Information

Data-In-situ Computing with One-Pixel-Multiple-Memristor Architecture for Neuromorphic Sequential Vision

Sun et al.

Supplementary Figures 1−14.

Supplementary Tables 1−4.

Supplementary Notes 1−4.

Supplementary Figures

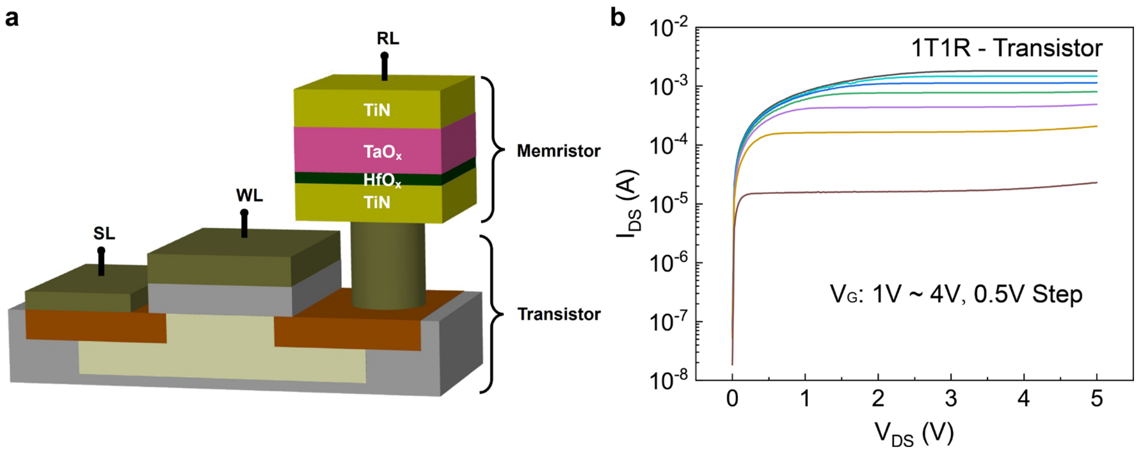

Supplementary Figure 1. The 1T1R structure and output characteristic of transistor. **a,** Schematic diagram of the structure connecting the transistor and the memristor. The memristor's bottom electrode is connected to the drain of the transistor. The top electrode of the memristor is connected to the RL of the 1T1R array, while the gate and source of the transistor are connected to WL and SL of the array, respectively. The transistor can provide current compliance to the attached memristor in resistance modulation and serve as a current switch to suppress current sneak-path in the 1T1R array. **b,** The output characteristic of the transistor in the 1T1R array. The transistor has different current compliance with various gate voltages (from 1 V to 4 V with 0.5 V step), which supports the attached memristor in resistance modulation.

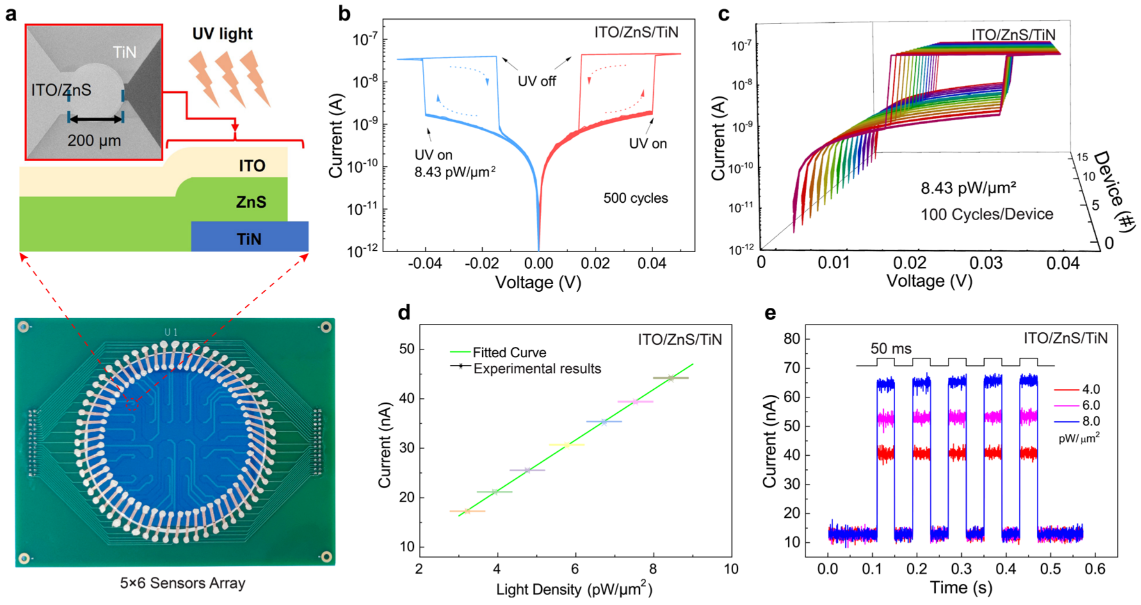

Supplementary Figure 2. Characterization of ITO\ZnS\TiN optical sensors. a, Schematic of the ITO\ZnS\TiN sensor and the SEM image of the fabricated device. In the experiment, a 5×6 sensor array is fabricated on a 4-inch wafer, the device pads are connected to a PCB board for optic and electrical tests. **b,** Quasi-DC characteristics of the sensor. An UV LED with a wavelength of 365 nm and intensity of 8.43 pW·μm^{-2} is used to investigate optoelectronic characteristic under quasi-DC tests. Both positive and negative voltage sweeps are performed 500 times, and the highly consistent curves indicate the great cycle-to-cycle (C2C) uniformity of the device. **c,** 100 cycles of 15 devices show great (device-to-device) D2D uniformity. **d,** Different light intensities are applied to characterize the sensing ability to light intensity, each is executed for 100 cycles. The responding current is plotted in box chart distribution. As the light intensity increases, the response gradually rises up. The mean current (I) of each group and the light intensity (D) can be fitted with power function relationship: $I = a{\cdot}D^b + c$. The green curve in the figure represents the fitting function, showing great coincidence with the measured results. **e,** The UV pulse test of the device, revealing fast responding ability for high-frame visual capture.

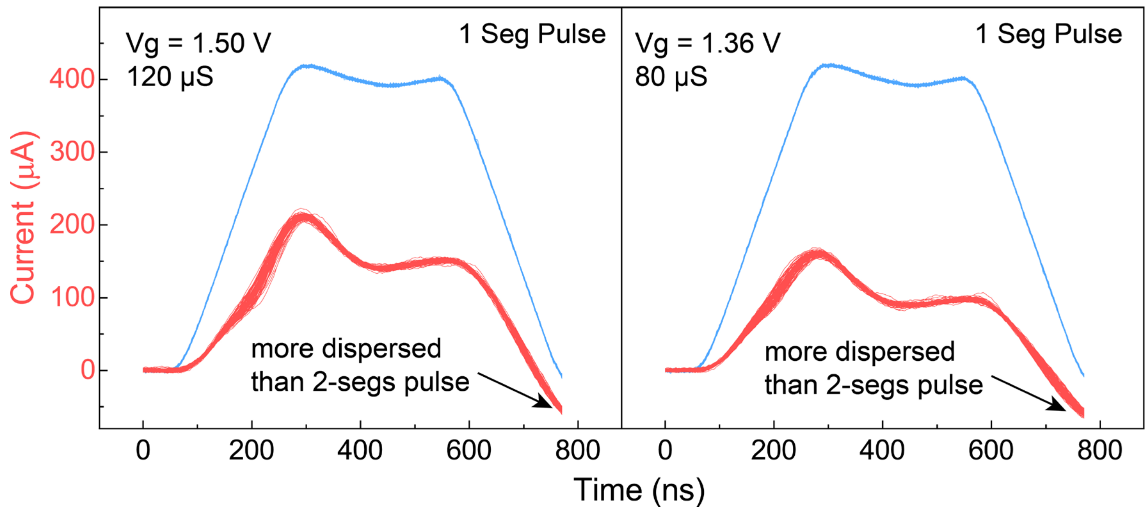

Supplementary Figure 3. Experimental results of OPM with one-segment pulse. 2 states are chosen as examples, and each is tested 50 times. At the falling edge of the pulse, the current curves are more dispersed than one-segment pulse, meaning increasing instability of the modulated device states.

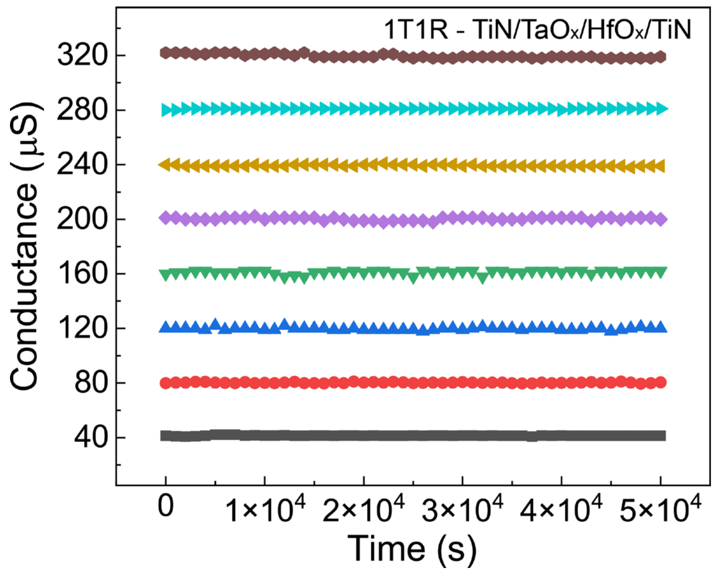

Supplementary Figure 4. Retention test of the 8 states after OPM modulation. The devices are modulated to 8 different states by OPM method and then monitored by 0.1 V reading bias for retention test. All the states can last 50000 s with only small fluctuation, revealing good device stability.

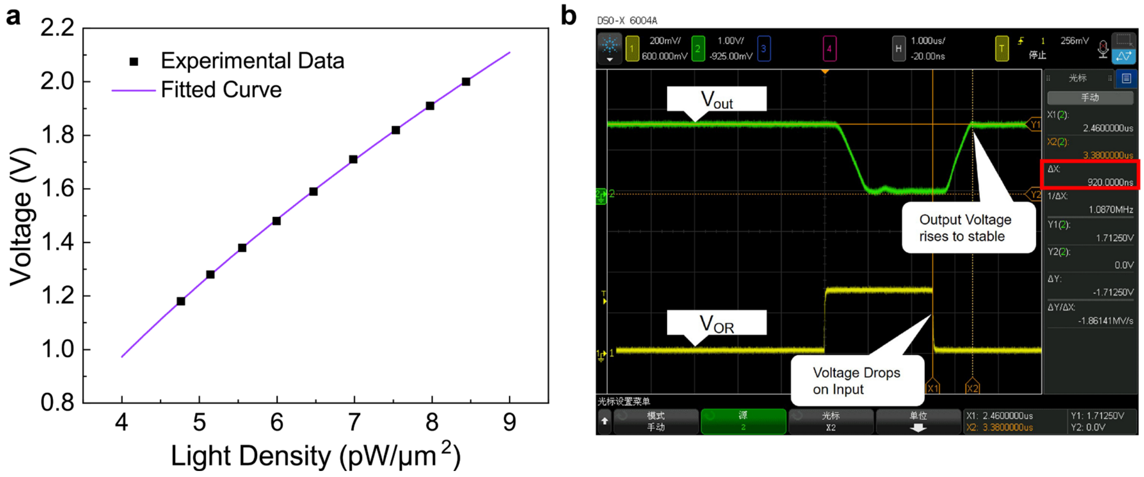

Supplementary Figure 5. The output characteristics of the transmission circuit. a, The relationship of the transmission circuit from light density to output voltage. The black spot is the measured data, and the purple curve is the fitted result with power function relationship: $I = a \cdot D^b + c$. The circuit process is purely linear, so the fitted relationship is consistent with the sensor's characteristic shown in **Supplementary Fig. 2d.** Furthermore, the output voltage of the circuit is adjusted to fit gate voltage of the 1T1R cell. **b,** The responding speed of the transmission circuit. A signal generator is connected to the input ports of the differential amplifier to provide the signal, while an oscilloscope simultaneously monitors the input and output signal of the circuit. The yellow curve represents the voltage division V_{OR} of the optical sensor, the falling portion is focused because V_{OR} drops under UV illumination. The green curve represents the output voltage of the differential circuit, which rises due to the decrease in the negative input. The results indicate that the time from the input voltage drop to the stabilization of the output voltage rise is about 920 ns, which reflects the speed of our conversion circuit. It should be noted that this delay is measured at the board level. With future chip-level integration, where parasitic capacitance and other non-ideal effects can be greatly reduced, we believe the circuit speed can be further improved.

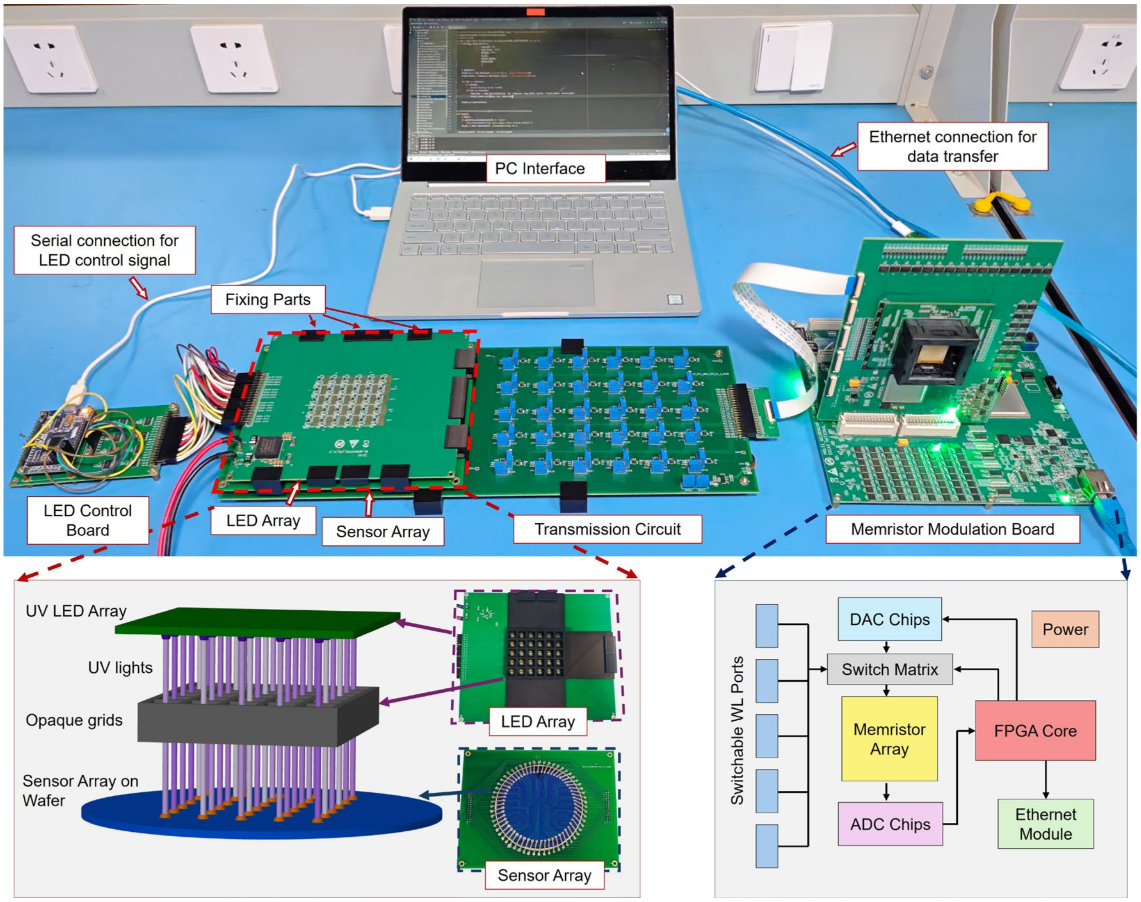

Supplementary Figure 6. Hardware implementation of the 1PnR architecture. The UV LED array board and UV sensor array board are positioned and fixed by one-to-one for light emitting and sensing per pixel. Opaque grids are applied to avoid cross-talk between light signals of different pixels. The 5×6 optical sensors are then connected to 30 channels of transmission circuit, which converts light information to gate voltages of memristor array for further image storage. The memristor modulation board mainly consists of FPGA core controller, array read-write circuits (ADC&DAC), WL connecting ports and switch matrix. Each WL port offers analogue connection to 30 channels of the 8k memristor array's WLs through switch matrix. And switch matrix can switch the connection of the memristor array' WLs between the WL ports for image exposure or DAC chips for data-in-situ computing.

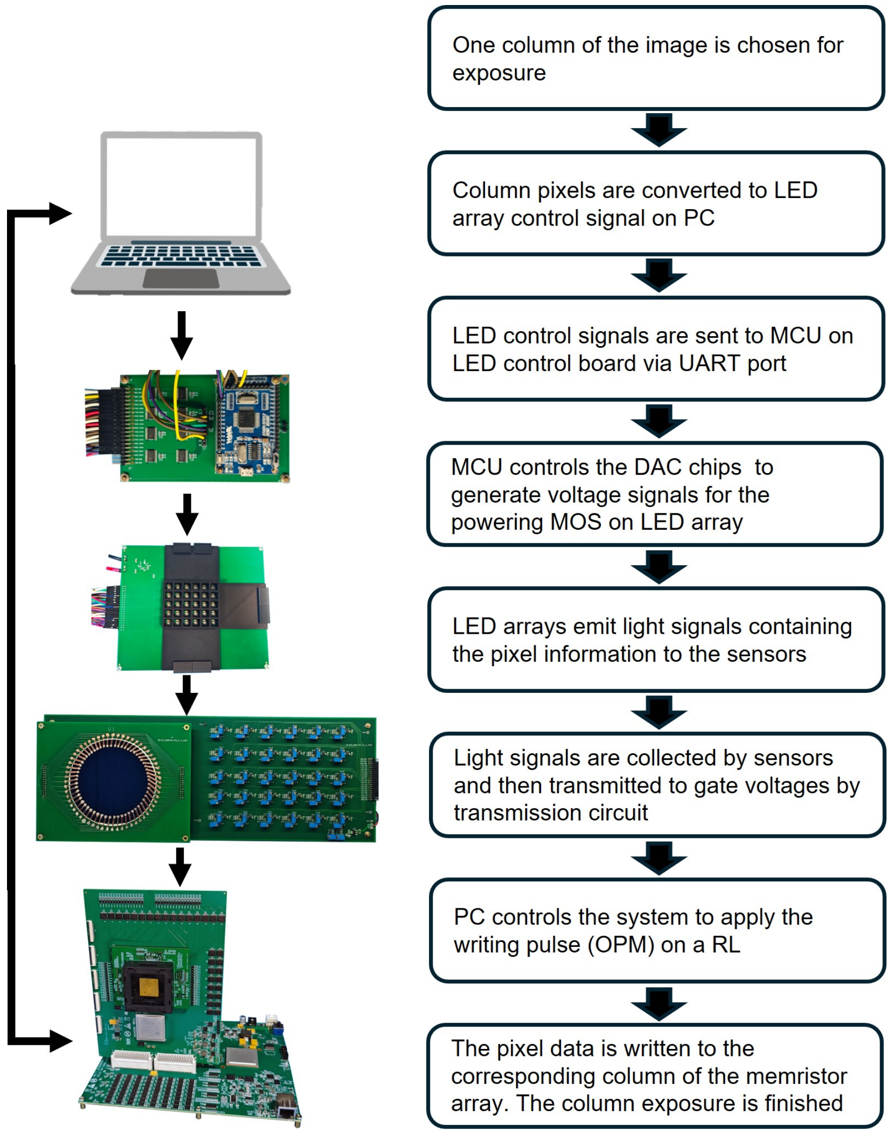

Supplementary Figure 7. The system timing sequence in image acquisition experiment. The PC host computer converts the grayscale values of the 30 pixels to be exposed into control voltages for the LED-driving MOS transistors. These values are transmitted via serial port to the MCU, which controls the DAC chips to output the corresponding voltage signals. Subsequently, the PC host computer sends the write signal to the memristor chip board system, the system then applies the One-Pulse-Method, enabling parallel writing of the 30-channel optical sensing information into the array.

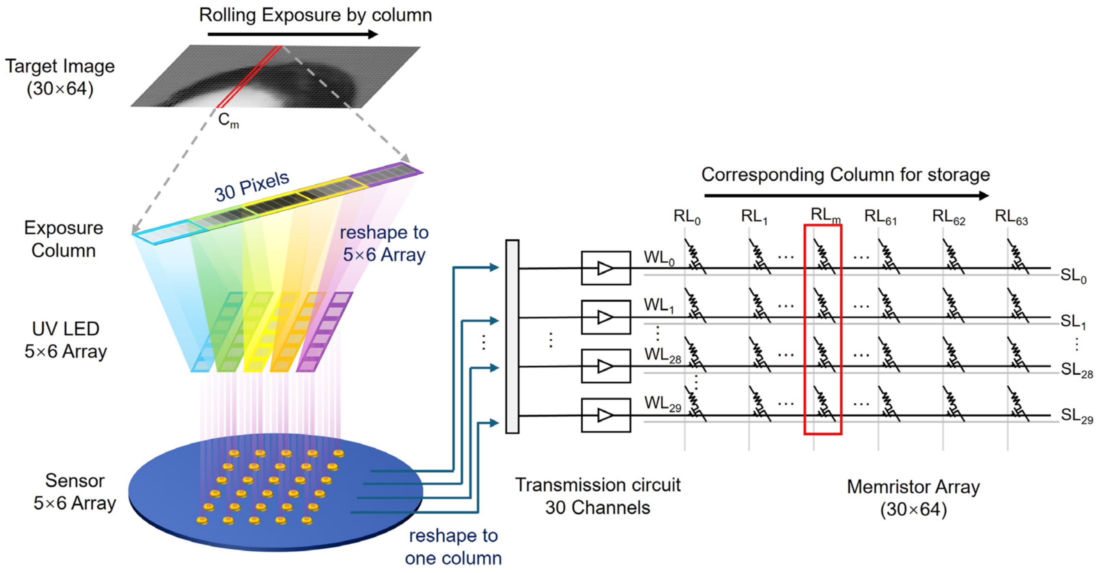

Supplementary Figure 8. The Schematic diagram of light collection mechanism for rolling exposure experiment. Each column of the target image is reshaped to 5×6 array for exposure experiment. The received signals are reshaped back to one column through the 30-channel conversion circuits and further written to the corresponding column of memristor array in parallel using the proposed One-Pulse Method. Then, next column of image is exposed and stored to the corresponding next column, till the whole image is sensed and stored.

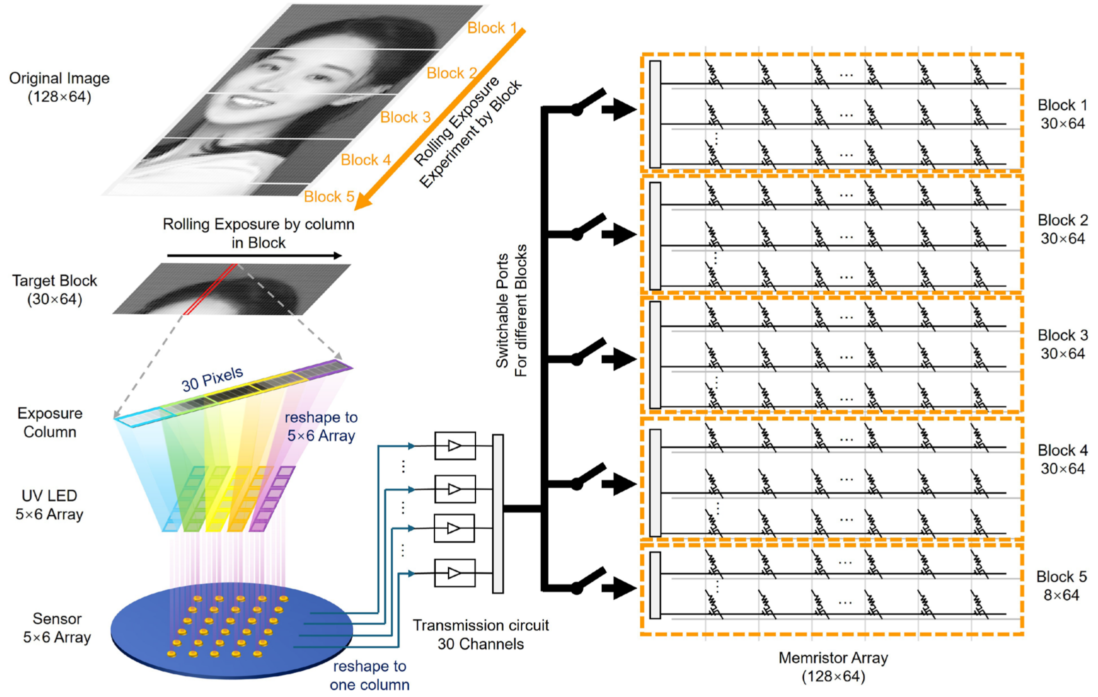

Supplementary Figure 9. The schematic of the experiments for portrait image. The image is divided into 5 blocks with 30 rows of pixels to match the 30-channel circuit prepared in the experiment, The rolling exposure of 30×64 is completed within each block first, and then the next block of image is exposed in sequence. Each block of image data is stored in the corresponding block region of memristor array through switchable ports in memristor modulation board.

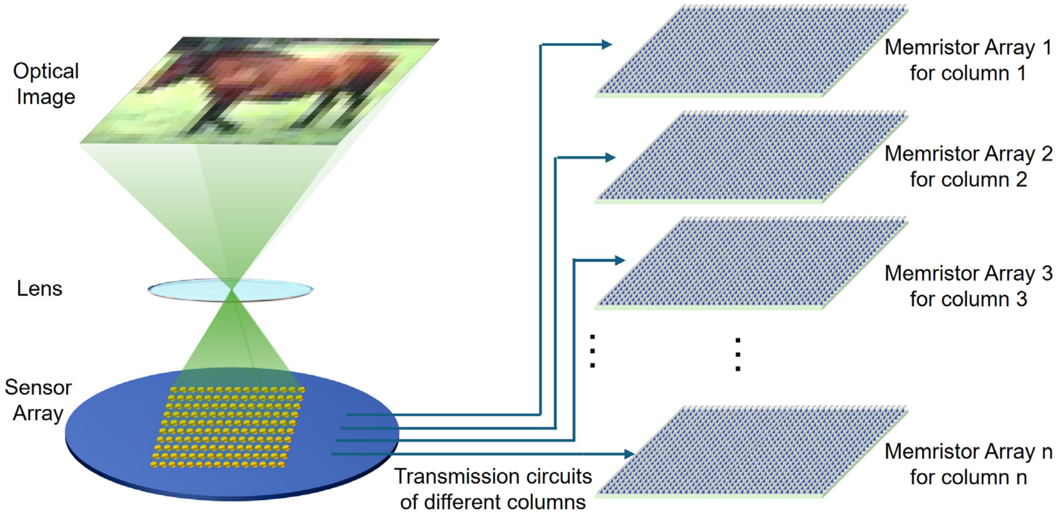

Supplementary Figure 10. Prospects of the 1PnR system for future image exposure. Multiple memristor arrays and large-scale sensors are employed, each column of sensors can be connected to a different memristor array. After exposure, writing pulses can be applied simultaneously to different arrays, enabling the entire image to be written into separate memristor arrays at the same time, offering very fast image exposure. The sample image is from CIFAR-10 dataset[15].

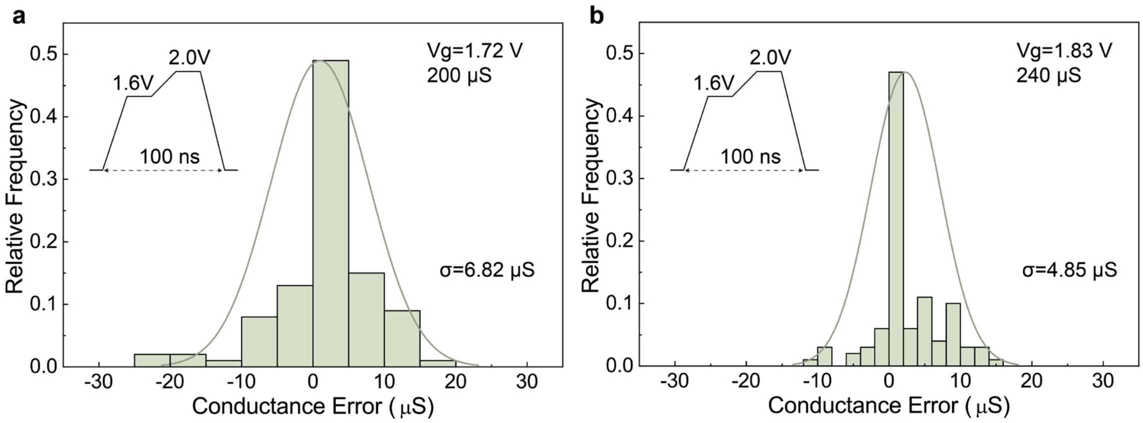

Supplementary Figure 11. The distribution of the conductance error using One-Pulse Modulation with 100 ns pulse width. Two conductance values 200 μS (**a**) and 240 μS (**b**) are selected as example for the high-speed modulation test. To fit the ultra-thin pulse width, pulse amplitudes of the 2 segments are raised to 1.6 V and 2.0 V, respectively. The gate voltages are also adjusted to 1.72 V and 1.83 V, respectively. The modulation results show that the OPM with a 100 ns pulse can achieve the same conductance accuracy as Fig. 2e, which demonstrates that the proposed 1PnR system can support ultra-fast exposure of optical image with 100 ns intervals.

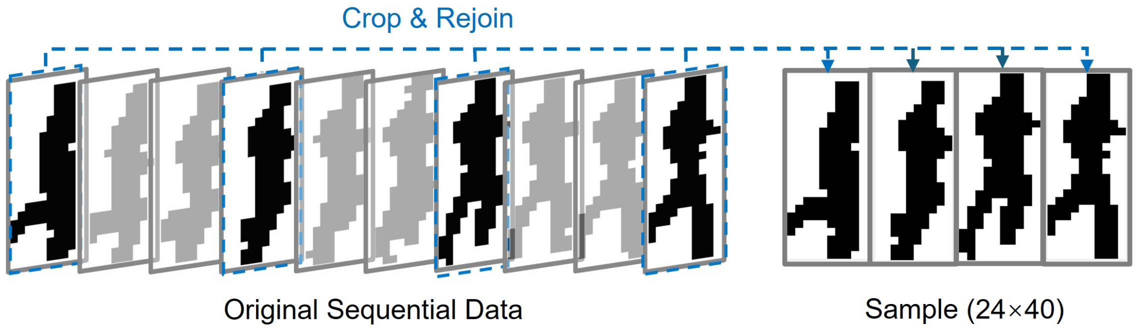

Supplementary Figure 12. The pre-process of the Weizmann Dataset used in network evaluation. The dataset has 10 actions, containing bend, jack, jump, pjump (jump in place), run, side, skip, walk, wave1 (in one hand) and wave2 (in two hands). Each action is performed by 9 people. The dataset is already binarized and aligned from video clips. In our simulation, the dataset is cropped to 24×10. To make sure that every sample (containing 4 frames) includes a whole period of periodic actions (such as walk, run and wave), the 4 frames in one sample are averagely picked from 12 consecutive frames in the video sequences.

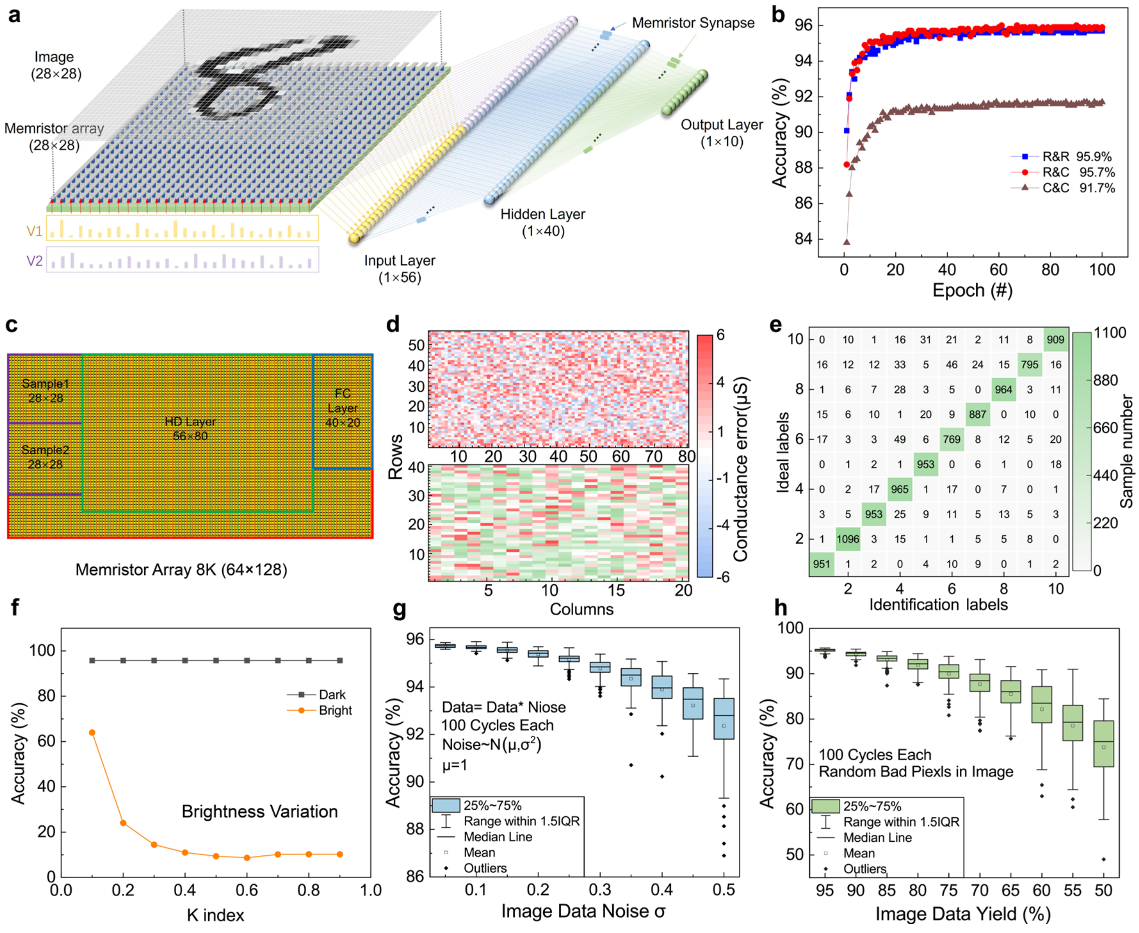

Supplementary Figure 13. Experimental demonstration of the MNIST recognition based on 1PnR hardware system. a, Schematic of the data-in-situ network for MNIST recognition. Two voltage weight vectors (VWV) are applied to perform data-in-situ computing, and 56×40×10 read-out layer is used for classification. **b,** Simulated results of the network. When 2 VWVs are both applied from the row of the memristor, the classification accuracy reaches best to 95.9%, if 2 VWVs are both applied from column of the array, the accuracy reduces to 91.7%. The MNIST samples have more features after data-in-situ computing in rows direction and achieve better classification performance. **c,** Deployment of the network in 8k memristor array. Color blocks reveal the partitions for layer mapping. **d,** Weight-transfer errors of the HD (56×40) and FC (40×10) layers to the memristor array by differential group. The transfer errors are limited within 6 μS by an array modulation script. **e,** Recognition results for MNIST dataset of the hardware system, with

92.42% accuracy. **f,** Simulation of the influence of image brightness on recognition accuracy. The grayscale values are mapped to the range [0, 1]. For over-dark variation, the grayscale values were compressed to [0, k], while for over-bright variation, they were compressed to [k, 1]. Over-dark variation has almost no effect on recognition accuracy, the sensor sensitivity should be sufficiently high. In contrast, over-bright variation has a significant impact on accuracy, due to the loss of dark-level information. **g,** Simulation of image sensor noise. The noise is applied in randomly generated normal distribution, with mean value of 1. Each standard deviation is simulated for 100 cycles. **h,** Simulation of image sensor yield rate, random bad pixels are applied according to the yield rate. It can be observed that the network maintains relatively good recognition performance at sensor noise of $\sigma = 0.2$ or a 90% sensor yield rate, revealing the good robustness of the data-in-situ network.

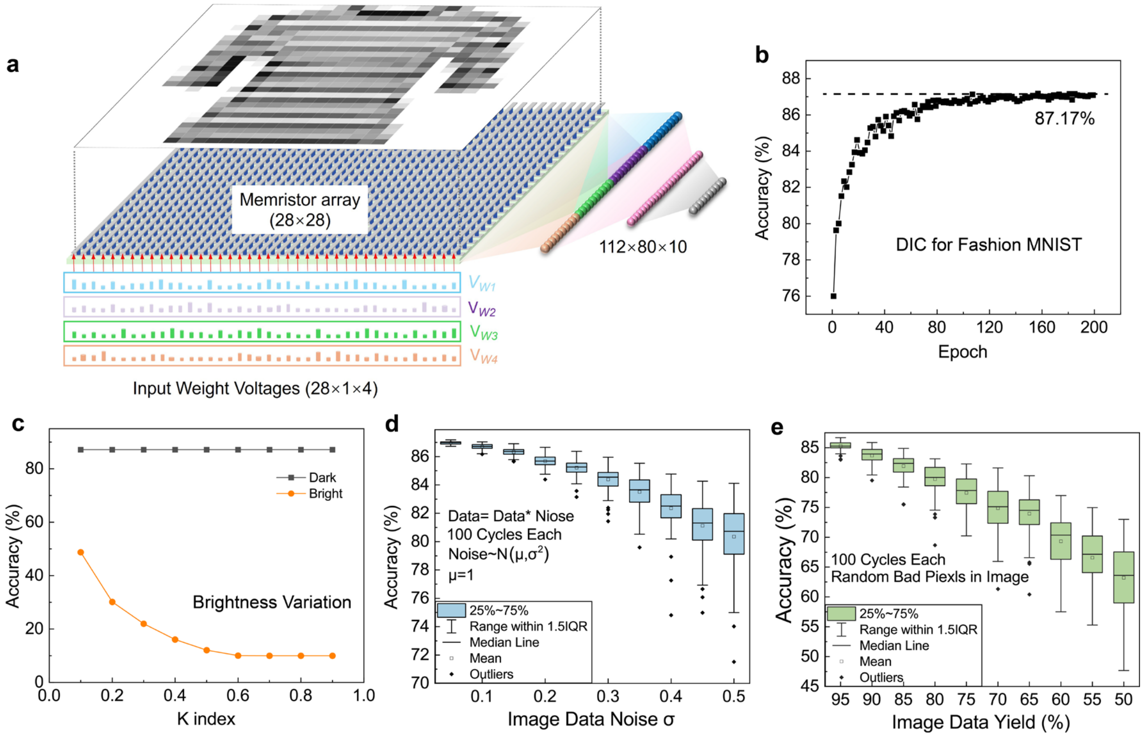

Supplementary Figure 14. Simulation of data-in-situ computing network for Fashion MNIST. a, Schematic diagram of the data-in-situ computing network for Fashion MNIST. Fashion MNIST has more complex features, 4 Voltage Vectors are applied to extract more image features, then attaching 112×80×10 network for further classification. **b**, Classification accuracy of the DIC network for Fashion MNIST. **c,** Simulation of the influence of image brightness on recognition accuracy. The grayscale values are mapped to the range [0, 1]. For over-dark variation, the grayscale values were compressed to [0, k], while for over-bright variation, they were compressed to [k, 1]. Over-dark variation has almost no effect on recognition accuracy, the sensor sensitivity should be sufficiently high. In contrast, over-bright variation has a significant impact on accuracy, due to the loss of dark-level information. **d,** Simulation of image sensor noise. The noise is applied in randomly generated normal distribution, with mean value of 1. Each standard deviation is simulated for 100 cycles. **e,** Simulation of image sensor yield rate, random bad pixels are applied according to the yield rate.

Supplementary Tables

Supplementary Table 1. Benchmark of image sensing

	ISSCC 2022[1]	JSSC 2023[2]	JSSC 2025[3]	This Work
Frame Rate (fps)	40	100	60	83333*
Pixel Array	120×120	320×320	512×320	120×120*
Power FOM** (pJ)	22.9	11000	8400	0.015
Pixel Resolution (bits)	8	10	10	Analog

* Assuming the image is 120×120, which requires 120 1PnR channels and 120 exposure operations. Each exposure operation can be finished by 100 ns pulse, so the equivalent frame rate is 1/(120×100e-9)≈83333 fps

** FOM is the energy per pixel per frame

Supplementary Table 2. Benchmark of 1PnR architecture and CMOS technology for 120×120 Image acquisition

	Sensing		Memory		Total	
	Delay	Energy	Delay	Energy	Delay	Energy
CMOS	25 ms	330 nJ	847 ns	156.67 nJ	~25 ms	~486.7 nJ
This Work	12 µs	0.216 nJ	0*	267.84 nJ	~12 µs	~268.1 nJ

* In 1PnR system, the sensing and storage is operated at the same, so the memory time delay can be considered as 0.

Supplementary Table 3. Comparison of the data-in-situ computing architecture for MNIST classification

	Network Structure	Input size	Network size	Image data Transmission	MNIST Accuracy	Evaluation Method
This work	Data-in-situ Computing	28×28	56×40×10	No	92.42%	Experiment
Nature. 2020[7]	CNN	28×28	C1:1×3×3×8<br>C2:8×3×3×12<br>FC:192×10	Yes	96.92%	Experiment
Science. 2023[5]	MLP	28×28	784×100×10	Yes	95.8%	Experiment
Science Advances. 2021[8]	MLP	8×8	64×64×10	Yes	93.63%	Experiment
Nature Nanotechnology. 2025[9]	MLP	28×28	784×300×10	Yes	96.1%	Simulation
Nature Communications 2024[10]	MLP	28×28	784×1102×64×10	Yes	97.1%	Simulation
Light: Science & Applications. 2025[11]	MLP	28×28	784×100×10	Yes	90.7%	Simulation

Supplementary Table 4. Comparison between the 1PnR architecture and existing sensor-memory integrated architecture

	Integrating Architecture	Verified Scale	Supported Network	Performance	Power Consumption	Exposure Time	Data Erase[a]	Data Transfer[b]
Adv. Mater. 2022[12]	1PT1R	16×3-PT	OANN	99.3%<br>("P" "K" "U")	/	/	Yes	No
Nat. Electron. 2024[13]	MPT1R	20×20-PT<br>36×32-1T1R	OCNN<br>ORNN<br>OSNN	91%<br>(MNIST)	/	/	Yes	No
Nat. Nanotechnol 2024[14]	1T1OR	128×8-1T1OR	ORNN	91.2%<br>(NTU-RGB dataset)	24.8 nJ[c]	/	Yes	Yes
This Work	1PnR	5×6-Piexl<br>64×128-1T1R	Data-in-situ Computing	95.7%<br>(Weizmann dataset)	581.73 pJ[d]	12 μs[e]	No	No

[a] Whether the storage device (memristor or optoelectronic memristor) needs to be erased before receiving next frame data in sequential vision.
[b] Whether the full image data needs to the transferred during computing process.
[c] The estimated energy consumption of the proposed OEM-based RC for one sample classification in human action recognition.
[d] The estimated energy consumption of the data-in-situ architecture for one sample in Weizmann dataset, which has about 160 times energy efficiency than typical CMOS systems.
[e] The estimated time consumption for processing 120×120 image using the 1PnR architecture, which exhibits ~2000 times reduction than CMOS sensors.

Supplementary Notes

Supplementary Note 1. The time/energy efficiency estimation and benchmark of the 1PnR system for image acquisition.

The time and energy efficiency of the proposed 1PnR system for image sensing and storage is estimated and compared with CMOS system. Correspondingly, the latest CMOS image sensors and DDR5 memory are used for image sensing and storage. 3 reprehensive works of reported CMOS sensor[1-3] are chosen for comparison, their parameters are listed in **Supplementary Table 1**. The latest reported DDR5 chip[4] is chosen for data storage, which is 1.36 pJ/bit and 8.5 Gbps on 16 DQ pins.

The CMOS sensors have different advances in time and energy, the one with balanced performance in work[1] is chosen for the comparison. To fit the sensor's parameter, the image size is assumed to be 120×120, which requires 120 channels and 120 exposure operations in the 1PnR system.

In the manuscript, the OPM pulse-width is set to 1 μs, which is limited by the board-level conversion circuit. Actually, the memristor can be modulated by OPM with 100 ns fast pulse, with consistent accuracy, as shown in **Supplementary Figure 11**. So, we believe the RES can be finished by 100 ns OPM with support of chip-level conversion circuit. And the equivalent frame rate can be estimated as 1/(120×100 ns) ≈83333 fps. According to the experimental results, the working voltage and current of the ITO/ZnS/TiN sensor is 0.3 V and 50 nA, respectively. So, the energy per pixel per frame (FOM) is estimated as 0.3 V×50 nA×100 ns=0.015 pJ.

Based on above parameters, the time and energy consumption for processing 120×120 image is then estimated. As for the CMOS system, the sensor's time delay can be considered as 1/40=25 ms, and its energy consumption is 13.2 μW/40 =330 nJ. The time delay of DDR5 chip for the 8-bit image storage can be estimated as 120×120×8 bit/(16 pins×8.5 Gbps)=847

ns, which is negligible compared to sensor's delay. And its energy consumption can be estimated as 120×120×8 bit×1.36 pJ≈156.67 nJ. Thus, the overall time delay can be estimated as 25 ms, and the energy consumption is 330 nJ+156.67 nJ≈486.7 nJ. As for the 1PnR system, the sensing and storage is operated at the same time, so the overall time delay can be considered as 100 ns×120=12 μs. The energy consumption of sensors is 0.015 pJ×120×120=0.216 nJ. The memristors are modulated by the One-Pulse-Modulation method for image storage. To estimate its energy consumption, the averaged voltage (1.12 V) of the OPM pulse and averaged memristor conductance (148.223 μS) after memorizing the example image are used. Thus, the energy consumption per one device can be estimated as 1.12 V^2×148.223 μS×100 ns=18.6 pJ. And the energy for storage 120×120 image is 18.6 pJ×120×120=267.84 nJ. So, the total energy consumption of the 1PnR system is 267.84 nJ+0.216 nJ≈268.1 nJ.

The estimation results are listed and compared in **Supplementary Table 2**. It can be concluded that the proposed 1PnR structure exhibits ~2000 times reduction in time delay and ~1.8 times reduction in energy consumption than CMOS technology.

Supplementary Note 2. The energy efficiency estimation and benchmark of the data-in-situ computing architecture.

To estimate the energy efficiency of data-in-situ computing architecture in future chip-level integration, the ADC and DAC parameters are chosen from a representative chip model in a reported work[5]. The memristive PE shape is 1152×512, integrating 512 ADCs and 1152 DACs. Operating with 30 ns pulse, the energy consumption per VMM calculation of ADC and DAC is 0.97 nJ and 2.54 nJ, respectively.

The data-in-situ computing for Weizmann human action dataset requires data-in-situ VMM calculation between 40×24 image and two 40×1 vectors, following a 48×8×10 perception network. The network is transferred to the memristor array using a differential strategy, the averaged power of single memristor device is estimated as 222.3 nW according to the average conductance and input voltage in array computation. Assuming the VMM pulse width is 30 ns, the energy consumption of data-in-situ part can be estimated as [222.3 nW×24×40×2+(0.97 nJ/512×48+2.54 nJ/1152×40)/30 ns]×2×30 ns ≈383.87 pJ. And the perception part is [222.3 nW×(48×16+8×20)+(0.97 nJ/512×36+2.54 nJ/1152×56)/30 ns]×30 ns≈197.86 pJ. Thus, the total consumption of the data-in-situ architecture for one sample in Weizmann dataset can be estimated as 383.87 + 197.86 = 581.73 pJ.

The energy consumption of CMOS system is estimated by a typical digital accelerator-based (HNPU-based) system[5,6], with DDR5 memory[4]. The power efficiency of the HNPU-based processer is 2.64 TOPS/W, and memory operating power of DDR5 memory is 1.36 pJ/bit. The on-board interconnect energy was assumed as 10 pJ/bit[5]. The total amount of VMM operations of the data-in-situ architecture for one sample in Weizmann dataset is 24×40×2+48×16+8×20=2848. Assuming the data is 8-bit, the memory fetching amount of the CMOS system is (24×40+48+8)×8=8128 bit. Thus, the power consumption of the processor can be estimated as (1/2.64TOPS/W)×2848= 1078.8 pJ, and that of memory is 11.36 pJ/bit ×7680 bit =92334 pJ. The total consumption of the CMOS system is 93413 pJ.

It can be concluded that the proposed data-in-situ computing architecture has about 160 times energy efficiency than typical CMOS systems.

Supplementary Note 3. Benchmark of the data-in-situ computing network for MNIST classification with other memristor-based works.

To further evaluate the performance of the data-in-situ computing network itself, benchmark is performed with the MNIST classification. Several representative works are chosen for comparison, and the results are listed in **Supplementary Table 3.** Our work requires the least input neurons and the smallest network scale for 28×28 full size MNIST dataset processing, as the data-in-situ computing performs the initial feature extraction of the image, significantly reducing the data that needs to be processed by the network. Furthermore, the experimental accuracy for MNIST classification reaches 92.42%, demonstrating the considerable classification ability of the data-in-situ computing network.

Supplementary Note 4. Comparison between the 1PnR architecture and existing sensor-memory integrated architecture

To highlight the innovations of the 1PnR architecture, 3 representative works about integrated sensing-storage-computation systems (1PT1R, MPT1R, 1T1OR architecture) are listed and compared in **Supplementary Table 4**.

Based on the comparative results with other works, the1PnR architecture can be identified the following advantages:

First, it supports continuous sensing and storage of sequential images. With one sensor corresponding to multiple storage units, sensing information can be rapidly and sequentially written into different storage units without erasing previously stored image data.

Second, it offers great scalability for large-scale integration. The architecture leverages relatively mature technologies for integrating optical sensors with memristor arrays. Since the sensors are directly connected to the gates of the memristor array, no modification of the internal structure of the memristor array is required, enabling straightforward integration. The image storage capacity can be easily expanded by simply increasing the scale of the memristor array.

Third, it provides high flexibility for system expansion. The independent nature of the sensor units and the memristor array allows for versatile configurations. For example, the sensor array can be integrated with multiple memristor arrays, where each column of sensors is connected to a different memristor array. After global exposure, writing pulses can be applied simultaneously to all arrays, enabling the entire image to be written at once and achieving ultra-high-speed image acquisition.

Supplementary References

1. Kim, S. et al. A Fully Digital Time-Mode CMOS Image Sensor with 22.9pJ/frame·pixel and 92dB Dynamic Range. *2022 IEEE International Solid-State Circuits Conference (ISSCC)*, 1-3, (2022).
2. Gulve, R. et al. 39 000-Subexposures/s Dual-ADC CMOS Image Sensor With Dual-Tap Coded-Exposure Pixels for Single-Shot HDR and 3-D Computational Imaging. *IEEE J. Solid-State Circuits* **58**, 3150-3163, (2023).
3. Luo, Y. et al. A 60-Frames/s CMOS Image Sensor With Pixelwise Conversion Gain Modulation and Self-Triggered ADCs for Per-Frame Adaptive DCG-HDR Imaging. *IEEE J. Solid-State Circuits* **60**, 568-578, (2025).
4. Ahn, H. -A. et al. A 1.01-V 8.5-Gb/s/pin 16-Gb LPDDR5x SDRAM With Advanced I/O Circuitry for High-Speed and Low-Power Applications. *IEEE J. Solid-State Circuits* **59**, 3479-3487, (2024).
5. Zhang, W. et al. Edge learning using a fully integrated neuro-inspired memristor chip. *Science* **381**, 1205-1211, (2023).
6. Han, D. et al. HNPU: An Adaptive DNN Training Processor Utilizing Stochastic Dynamic Fixed-Point and Active Bit-Precision Searching. *IEEE J. Solid-State Circuits* **56**, 2858-2869, (2021).
7. Yao, P. et al. Fully hardware-implemented memristor convolutional neural network. *Nature*. **577** 641–646 (2020).
8. Kiani, F. et al. A fully hardware-based memristive multilayer neural network. *Sci. Adv.* **7**, eabj4801 (2021).
9. Huang, H. et al. Fully integrated multi-mode optoelectronic memristor array for diversified in-sensor computing. *Nat. Nanotechnol.* **20**, 93–103 (2025).
10. Jebali, F. et al. Powering AI at the edge: A robust, memristor-based binarized neural network with near-memory computing and miniaturized solar cell. *Nat. Commun.* **15**, 741 (2024).
11. Cui, D. et al. Versatile optoelectronic memristor based on wide-bandgap Ga_2O_3 for artificial synapses and neuromorphic computing. *Light Sci. Appl.* **14**, 161 (2025).
12. Dang, B. et al. One-phototransistor–one-memristor array with high-linearity light-tunable weight for optic neuromorphic computing. *Adv. Mater.* **35**, 2204844 (2023).
13. Dang, B. et al. Reconfigurable in-sensor processing based on a multi-phototransistor–one-memristor array. *Nat Electron* **7**, 991-1003 (2024).
14. Huang, H. et al. Fully integrated multi-mode optoelectronic memristor array for diversified in-sensor computing. *Nat. Nanotechnol.* **20**, 93-103 (2025).

15. Krizhevsky, A. Learning Multiple Layers of Features from Tiny Images. (2009).
