# Supplementary material for: Data-In-situ Computing with One-Pixel-Multiple-Memristor Architecture for Neuromorphic Sequential Vision
Source: Nat Commun. 2026 Mar 19;17:4244. doi: 10.1038/s41467-026-70860-y (PMC13168392; doi:10.1038/s41467-026-70860-y)
Supplement: Supplementary file 2 — Editor Summary [file 41467_2026_70860_MOESM2_ESM.docx]

Conventional neuromorphic vision systems based on pixels-to-one-memristor architecture face efficiency challenge. Here, Sun et al. report an architecture employing one-pixel-to-multiple-memristor structure, mapping weight updates to voltage vectors to enhance both processing speed and energy efficiency.
